# Immediate and Long-term Complications of Direct-to-implant Breast Reconstruction after Nipple- or Skin-sparing Mastectomy

**DOI:** 10.1097/GOX.0000000000001977

**Published:** 2018-11-05

**Authors:** Thomas C. Lam, Frank Hsieh, James Salinas, John Boyages

**Affiliations:** From the * Department of Plastic & Reconstructive Surgery, Westmead Hospital, Sydney, NSW, Australia; † Faculty of Medicine and Health Sciences, Macquarie University, Sydney, Australia.

## Abstract

**Background:**

Traditionally, breast reconstruction options after mastectomy comprise an autologous flap or staged expander/implant reconstruction, or a combination of both. Recent introduction of skin or nipple-sparing mastectomies have led to much interest in direct-to-implant immediate breast reconstructions. We performed a retrospective review of our initial experience.

**Methods:**

Between June 1998 and December 2010, 31 of 671 patients (4.6%) who received implant-only breast reconstruction underwent direct-to-implant immediate breast reconstruction after mastectomy for primary or recurrent cancers, or risk reduction. Their files were audited, and the primary factor examined was the failure of reconstruction with loss of prosthesis. Other complications, revision surgery, and aesthetic result are also recorded.

**Results:**

The mean follow-up period for the 31 patients was 49.5 months. A total of 45 mastectomies were performed for 21 primary and 4 recurrent breast cancers after previous conservation surgery and radiotherapy (RT), and 20 for risk reduction. Ten patients received RT (4 before mastectomy and 6 afterward). Average size of implants was 380.0 g (range, 205–620 g). The most common postoperative complications were seromas (20%); only 1 implant was lost (2.22%). Nineteen breasts required revision surgery after 6 months with 1 more implant lost. Despite the high revision rate, 28 (90.3%) had excellent or good aesthetic result.

**Conclusions:**

Immediate single-stage direct-to-implant breast reconstruction has a high rate of both immediate postoperative complications and revisions after 6 months, especially after RT. However, most complications are manageable and do not necessarily result in implant loss. Most cases can have a successful outcome without implant loss with excellent or good cosmetic results.

## INTRODUCTION

Since the temporary tissue expander was introduced by Radovan^1^ in 1982, two-stage prosthetic breast reconstruction comprising initial insertion of a tissue expander followed by exchange to an implant after a period of inflation has become a well-established option of breast reconstruction either immediately after mastectomy or later.^2^ Most of the current literature concentrates on the former, where the incidence has risen rapidly, especially in the United States.^2–7^ However, despite the connotation of the word “immediate,” patients usually wake from their mastectomy with a less than half filled tissue expander due to the tight submuscular pocket. The final reconstruction result is not achieved for at least 3 months and usually much later, especially if adjuvant chemotherapy and/or radiotherapy (RT) is required. This delay is often a source of distress to patients.

More recently, however, there has been a concerted push toward skin-sparing mastectomy (SSM) or nipple-sparing mastectomy (NSM),^8–11^ whereby the first stage of tissue expansion is by-passed and an implant inserted immediately under the adequate skin envelope. The patient wakes from the anesthetic with the reconstruction completed, a true immediate breast reconstruction.^12^

We have been regularly performing single-stage direct-to-implant (DTI) immediate breast reconstruction after SSM or NSM in these patients since commencement of these types of mastectomies by our breast surgeons as a routine since 2008. This retrospective study examines the immediate and long-term outcome of this initial group of patients with or without postmastectomy RT.

## PATIENTS AND METHODS

A retrospective review was undertaken of all breast reconstructions performed by a single surgeon (T.L.) between June 1998 and December 2010. A total of 671 patients who received prosthetic breast reconstruction were identified. Of these, 35 patients underwent SSM or NSM and/or single-stage DTI reconstructions with anatomical silicone gel implants. Three of these patients who underwent delayed single-stage implant reconstruction or an immediate 2-stage reconstruction were excluded. As a result, 31 (4.6%) patients were available for the current study.

### Surgical Technique

Our breast surgeons have used several incisions in performing NSM that include a lateral transverse incision with or without a superior or inferior periareolar extension, an infra-mammary crease^13^ or infra-areolar vertical incision. Occasionally, a “gull-wing” skin pattern was removed superior to the nipple-areola complex (NAC) to raise the nipple position. For SSM, the most common incision is a transverse ellipse removing the NAC. A “Wise-pattern” breast reduction incision can also be performed as an NSM or SSM with the use of a de-epithelialized inferior mastectomy flap^14–16^ sutured to the freed inferior border of the pectoralis major muscle to cover the lower pole of the implant. This method results in a smaller reconstruction than the original breast size and is often performed bilaterally for patients otherwise desiring a breast reduction.

Implants were inserted subcutaneously, or more commonly subpectorally, extending to a subcutaneous pocket infero-laterally, or continued under the serratus anterior fascia. No acellular dermal matrix (ADM) was used in this early group of patients. Only anatomical silicone gel breast implants were used (Allergan or Mentor). The final implant weight was guided by preoperative breast measurements and the specimen weight intraoperatively. A drain is inserted subcutaneously and another one is inserted under the pectoralis major muscle if the implant is inserted subpectorally. The drains are removed once the drainage volume falls below 30 cc over a 24-hour period postoperatively. Oral antibiotics are given until the drains are removed. Once the final pathology report is available, a decision is made about the necessity of adjuvant chemotherapy and/or RT.

The primary factor examined in this study is failure of the reconstruction from implant loss and other postoperative complications, and late complications requiring surgical revision after 6 months. Aesthetic results were also recorded and assessed with a 4-point scale ranging from poor to excellent by the author (T.L.). “Excellent” refers to a good reconstructed breast shape and symmetrical to the contralateral breast. A “good” result is a good shape but not symmetrical to the contralateral breast, although a “fair” result is an average-looking reconstructed breast mound, which is not symmetrical to the contralateral breast. Reconstruction failure is assessed as “poor.” Patients were not asked to rate their implant, as they tend to score higher than a physician.^17^ We believe that by defining the common subjective terms of “excellent/good/fair/poor” made the assessments more objective. Furthermore, individual patients rather than individual breast were scored for cosmesis.

This study was approved by the Western Sydney Local Health Network Human Research Ethics Committee.

## RESULTS

Of the 31 patients in this study, 10 (32.3%) had previous RT including 4 with recurrence in the breast after previous conservative surgery, and RT and 6 received postmastectomy RT following their initial diagnosis of breast cancer. One of the latter 6 patients also underwent a contralateral mastectomy for bilateral primary breast cancers, but only received radiation to 1 side. A further 3 patients had a contralateral risk-reducing mastectomy (RRM). The remaining 21 patients did not receive radiation, 9 of whom also had a contralateral RRM and another one had bilateral breast cancers, giving a total of 14 bilateral breast reconstruction cases resulting in 45 breast reconstructions.

The overall average age was 49.4 years (range, 26.1–64.9) and average follow-up was 49.5 months (range, 3–79). There was no significant difference between the 2 groups, although patients who did not receive RT tended to be younger and the average follow-up was longer (54.6 months) compared with the RT group (38.7 months; Table 1).

**Table 1.**
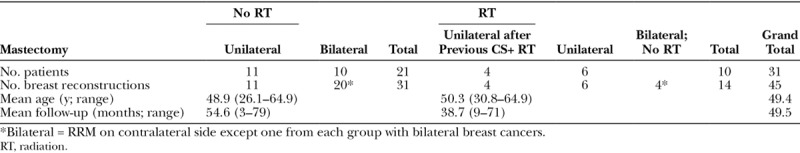
Patient Demographics

To delineate the risk of each breast reconstruction more accurately, we divided the breast reconstructions according to their RT status; group 1 (n = 35) includes the total number of breast reconstructions, which did not receive pre- or postmastectomy adjuvant RT (31 from nonirradiated patients and 4 contralateral breast reconstructions from patients who received RT to one breast), and group 2 comprised 10 unilateral breast reconstructions, which received RT pre- or postoperatively. To assess any possible difference between pre- and postmastectomy RT effects, group 2 was further subdivided into group 2A, consisting of the 4 patients who initially had conservative surgery and RT and treated with a mastectomy for a recurrence in the breast, and group 2B, comprising the remaining 6 patients who were initially treated with postmastectomy RT. The pathology and surgery data are recorded in Table 2.

**Table 2.**
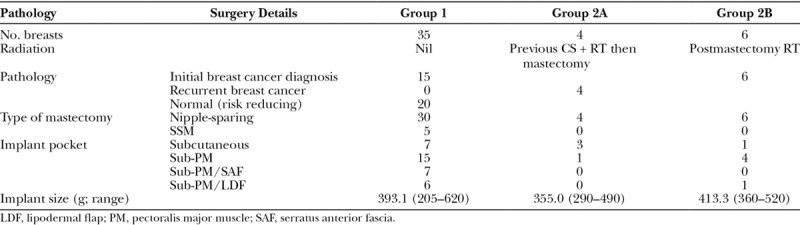
Pathology and Surgery Details

Most patients underwent NSM (40/45, 89%) and DTI reconstruction in a subpectoral pocket alone, with the serratus anterior fascia, or a de-epithelialized lipodermal flap. The average breast implant size for group 1 was 393.1 g, similar to group 2 at 390.0 g. However, those with premastectomy RT tended to have a smaller implant (group 2A) than those who received postmastectomy RT (group 2B; Table 2).

### Postoperative Complications Including Loss of Implant

Overall, only 1 implant was lost (1/45 = 2.2%) from postoperative wound infection (Table 3). This occurred in group 2A where the patient had recurrent breast cancer after previous conservation surgery and RT. The most common was a seroma of which there were 7 in group 1 and one each in groups 2A and B. Partial nipple necrosis after nipple-sparing mastectomy was also common, with 7 in group 1 and another in group 2B. One further patient in group 2B had a total NAC necrosis with a Wise-pattern NSM requiring debridement and full thickness skin graft. Another patient in group 1 returned to theater for debridement of delayed wound healing. Apart from the patient who lost her implant after a major wound infection (defined as requiring intravenous antibiotics), there were 2 other major wound infections, one each in Groups 2A and B, both settled with intravenous antibiotics. In total, there were 15 postoperative complications arising from group 1 (42.8%) and 7 from group 2 (70%). The patient who lost her implant later had a free transverse rectus abdominis myocutaneous (TRAM) flap with good result.

**Table 3.**
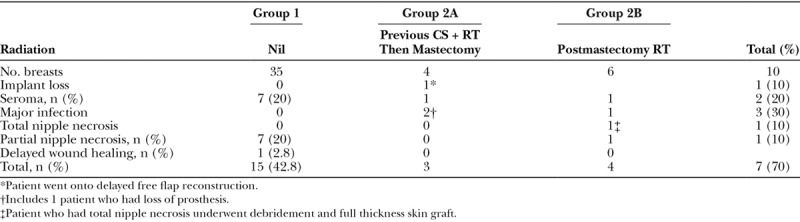
Postoperative Complications within 6 Months

### Late Complications Requiring Revision Surgery after 6 Months

With an average follow-up of 48.4 months, there were 19 breast reconstructions (42.2%) with late complications requiring revision surgery 6 months after mastectomy and reconstruction (Table 4). Rotation or displacement of the implant was the most common, recorded in 7 patients. Capsular contracture was the next most common, in 4 patients. One of the 2 patients from group 2B elected to convert to a free TRAM flap. Another patient in group 2B developed a late seroma 18 months after completing postmastectomy RT. The implant was changed and this was complicated by a seroma that became infected. The implant was eventually removed. She underwent further reconstruction with a latissimus dorsi myocutaneous flap only. Hence, a total of 2 implants were lost including the one postoperatively (2/45 = 4.4%).

**Table 4.**
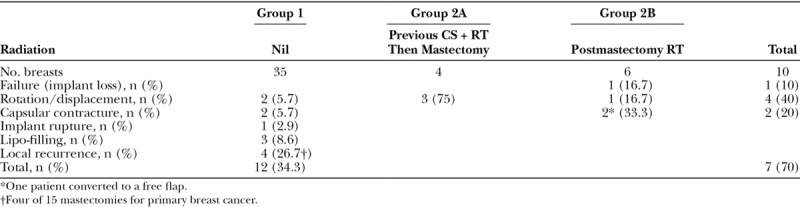
Late Complications Requiring Revision Surgery after 6 Months

One patient from group 1 with a contralateral RRM developed implant rupture 5 years later and the implant was replaced. Three patients in group 1 developed visible skin rippling and underwent 7 sessions of lipo-filling.

There were 4 local recurrence of cancers in group 1 (4/15 mastectomies for primary cancer without adjuvant RT = 26.7%) but none from group 2. One patient developed chest wall recurrence 32 months after NSM requiring local resection followed by chemotherapy and RT and is disease-free 42 months later. Another patient developed Paget’s disease of the nipple 51 months after NSM and had the NAC resected followed by RT and is disease-free 15 months later. A further patient with bilateral breast cancers developed local recurrence in her right breast biopsy track 22 months later that was treated with local excision and RT. She then developed local recurrence in the left NAC 56 months from her original surgery requiring resection of the NAC followed by chemotherapy and RT. She is disease-free 23 months later.

### Aesthetic Result

The final aesthetic result was clinically assessed per patient (Table 5). Of the 21 patients in group 1, 10 were judged as excellent (47.6%) and the remaining 11 good (52.4%). In group 2, three patients underwent further reconstructions. One of the 2 who have lost their implants from infections underwent a TRAM flap with a good result and the other with a LD flap with a fair result. The third patient underwent a TRAM flap after an elective removal of her implant due to capsular contracture and had an excellent result. However, since the aesthetic result is assessed in relation to the implant reconstruction, these further reconstructions are excluded in the assessment, and these 3 patients are assessed as 2 poor, 1 fair leaving a total of 28 patients assessed as excellent or good (28/31 = 90.3%).

**Table 5.**
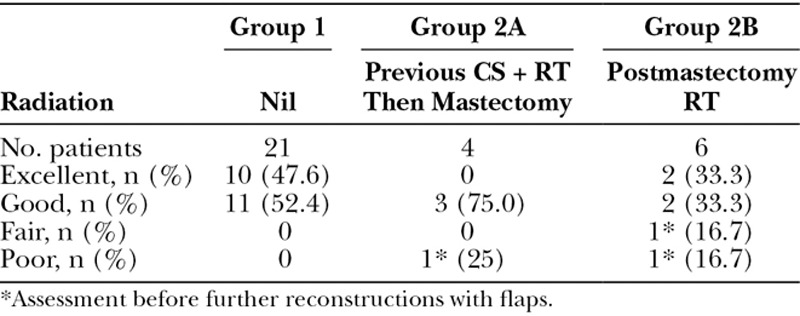
Aesthetic Results Per Patient

## DISCUSSION

Prosthetic breast reconstruction has come full circle. After the first silicone breast implant was introduced in 1962,^18^ prosthetic breast reconstructions consisted mainly of delayed single-stage insertion of an implant. The implant size was usually limited by the tightness of the skin after a radical or modified-radical mastectomy.^19,20^ Consequently, the reconstructed breasts were often asymmetrical to the contralateral breast. Subsequent development of temporary saline-filled tissue expanders^1^ and more recently patient-controlled release of compressed CO_2_ AirXpanders^21^ allowed gradual stretching of the skin envelope to accommodate, at a second operation, a much larger implant, providing a better match to the contralateral breast. As a result, 2-stage expander/implant breast reconstruction has become the most common breast reconstruction technique globally.^22–24^ However, more recently, with the introduction of first “SSM”^8,25,26^ and then “NSM,”^9–11,27,28^ “DTI” breast reconstructions are regaining popularity.^12,29^

**Fig. 1.**
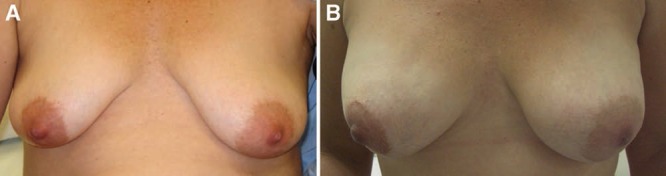
A 41-year-old with risk-reducing nipple-sparing mastectomies and subcutaneous implant reconstructions: (A) preoperative; (B) 18 months postoperative.

Apart from patients with advanced breast cancer or cancers located near the NAC, NSM is becoming more common than SSM, now extending to recurrent breast cancer after previous conservation surgery and RT, as in our group 2A patients. In our current study, most patients underwent an NSM, which is probably the ideal operation for RRM without breast cancer^30^ (Fig. 1).

With NSM, we find that placing an anatomical gel implant in a subcutaneous pocket is simplest, replacing the removed breast tissue and giving an excellent immediate result. However, skin flaps after mastectomy are often thin with questionable viability, resulting in flap necrosis. This has been reported as a common complication usually resulting in implant loss.^31^ In addition, the skin envelope is stretchable, especially when complicated by a seroma, and implant displacement can occur. Thirdly, once swelling settles and the skin envelope and capsule shrink around the implant, it may show implant rippling. As a result, we have moved our implant pockets subpectorally as is commonly reported.^2^ However, since the pectoralis muscle is not attached superficially to the breast skin, once it is freed from its insertion inferiorly, the muscle contracts superiorly leaving only adequate cover to the upper half of the implant. In this series of our early experience, we were reluctant to use any ADM as it introduces another “implant” with its accompanying risk of added complications, including the “red-breast syndrome,” which may also result in implant loss.^32–34^ Our results show that DTI reconstructions can be achieved without using ADMs. However, it may not be generalizable to ADM-assisted DTI cases, which has become more popular. When a patient with large pendulous breasts wishes to reduce their size, a Wise-pattern skin reduction and an inferior de-epithelialized lipodermal flap is ideal, as it utilizes an autologous “mesh” without risk of an ADM.^14–16^ In this series, we have 7 such patients and as such, the size of implants used were relatively smaller. Interestingly, recent reports have again placed the implant subcutaneously, but with total or anterior ADM cover.^35–37^ The ADM is sutured to the chest wall, restricting implant displacement and relieving the lower skin flap of the implant weight. Any subsequently visible rippling can be managed by lipofilling.^38^

### Radiation Therapy

It is well known that RT after breast augmentation and subsequent lumpectomy for cancer commonly results in higher incidence of severe capsular contractures, even with relatively good cover of remaining breast tissue.^39,40^ With the recent broadening of indication for adjuvant RT after mastectomy, patients are increasingly receiving postmastectomy RT.^41,42^ As these patients after DTI immediate breast reconstructions have an implant without the thicker natural covering of breast tissue after mastectomy, the incidence of severe capsular contracture is expected to be even higher. However, the need for RT may not be apparent before mastectomy and by the time the pathology report is available postoperatively, the implant is already in place. These patients may require revision later due to severe capsular contractures, which occurred in one-third of our patients (Table 4).

Traditionally, the best option for these patients is an autologous breast reconstruction such as the TRAM or deep inferior epigastric perforator flaps.^43^ However, increasingly we are dealing with nulliparous younger or slim patients who do not have excess lower abdominal soft tissue for such a flap. Apart from the abdominal wall, there are other free flap donor sites^44,45^ that have been reported, but not all patients are willing to sacrifice another part of their body as a donor area with resultant scars.^46,47^ Other options include the “pedicled” latissimus dorsi flap, often used together with an expander or implant when autologous options are excluded.^48^ Otherwise, revision of the breast reconstruction with extensive capsular release can be tried, possibly with a slightly larger implant.

In this study of our early experience of DTI, immediate breast reconstruction after NSM or SSM, we have analyzed our results per breast, which we believe reflects our results more accurately. Previously, most reports analyzed results per patient and as most had unilateral mastectomies for cancer, the number of patients and breast reconstructions were similar. However, prophylactic contralateral RRMs have become more commonplace. In addition, with the detection of breast cancer genes, high-risk patients are undergoing bilateral RRM without having breast cancer, such that this group has the fastest increase in incidence and DTI reconstruction.^2^ This accounted for 5 of 31 (16.1%) patients. Another 10 (32.3%) unilateral breast cancer patients underwent contralateral RRM in our study.

It has been generally accepted in the literature that implant-only breast reconstruction is contraindicated after RT.^10,27^ Lam et al.^49^ published a systematic review of immediate 2-stage prosthetic breast reconstruction in 715 patients who underwent adjuvant RT after insertion of a tissue expander or an implant and found an average reconstruction failure rate of 18.6% (range, 0–45%). Interestingly, it was also found that prosthesis loss was higher if adjuvant RT was given to the tissue expander rather than the implant after stage 2, a situation not dissimilar to DTI breast reconstruction. Despite that relatively high failure rate, most authors continued to recommend immediate breast reconstruction after mastectomy and RT.

Although this series of our early experience is relatively small, this is a series of 31 consecutive patients who underwent DTI breast reconstruction after NSM or SSM with or without RT. Encouragingly, we have successfully completed reconstruction in over 93% of patients. In addition, there was no difference in implant size for primary breast cancer patients, although in previously irradiated patients implant size is around 15% smaller than the average in patients who did not receive RT after mastectomy. However, the rate of late complications requiring revision surgery after 6 months is very high, 34.3% in group 1 and 70% in group 2.

### Local Recurrence

In a recent “comprehensive review of the literature,” Mallon et al.^11^ found that the average rate of occult nipple malignancy was 11.5% (range, 0–53%), suggesting some sampling is important in NSM. The overall incidence of NAC recurrence was 0.9%, and incidence of skin flap recurrence was 4.2%. However, mean follow-up was only 38.4 months. This is lower than our cohort, but our numbers are small. On the other hand, our results showed that when reporting local recurrence, it is critical to calculate per breast rather than per patient, as we had 1 patient with bilateral breast cancer who developed local recurrence in each breast at different times. In addition, many patients undergoing NSM do not have breast cancer and should be excluded from the denominator. Hence, we had 4 local recurrences in 15 patients (26.7%) who underwent NSM after mastectomy for primary breast cancer without adjuvant RT. This high rate is concerning but our breast surgical colleagues have subsequently published 87 patients with 118 NSM including most of our cohort have reported a 7% local recurrence rate,^50^ quoted as “consistent with other published data, which report local recurrence rates between 0% and 12%.” In the same review by Mallon et al.,^11^ it was noted that overall incidence of full-thickness nipple necrosis was 2.9% and for partial-thickness loss, 6.3% which is consistent with our findings.

Overall, we found that aesthetic results were excellent or good despite a high revision rate, including after postmastectomy RT. With recent interests in fat transfer, we have performed several revisions with lipofilling and anticipate that with experience, final aesthetic results can be further improved.^38^

## CONCLUSIONS

Single-stage DTI immediate breast reconstruction after SSM or NSM may have a high complication and revision rate, but most can still achieve excellent or good results, including after RT. Long-term follow-up studies are required to assess the revision rate and local recurrence.
